# Measurement properties of painDETECT: Rasch analysis of responses from community-dwelling adults with neuropathic pain

**DOI:** 10.1186/s12883-017-0825-2

**Published:** 2017-03-04

**Authors:** Tara L. Packham, Joseph C. Cappelleri, Alesia Sadosky, Joy C. MacDermid, Florian Brunner

**Affiliations:** 1 School of Rehabilitation Sciences, McMaster University, 1400 Main St. W., Hamilton, ON L8S 1C7 Canada; 2 Pfizer Inc, Groton, CT USA; 3 Pfizer Inc., New York, NY USA; 4 School of Physical Therapy, Western University, London, Ontario Canada; 5 Department of Physical Medicine and Rheumatology, Balgrist University Hospital, Forchstrasse 340, CH-8008 Zurich, Switzerland

**Keywords:** Neuropathic pain, PainDETECT, Outcome measurement, Rasch analysis

## Abstract

**Background:**

painDETECT (PD-Q) is a self-reported assessment of pain qualities developed as a screening tool for pain of neuropathic origin. Rasch analysis is a strategy for examining the measurement characteristics of a scale using a form of item response theory. We conducted a Rasch analysis to consider if the scoring and measurement properties of PD-Q would support its use as an outcome measure.

**Methods:**

Rasch analysis was conducted on PD-Q scores drawn from a cross-sectional study of the burden and costs of NeP. The analysis followed an iterative process based on recommendations in the literature, including examination of sequential scoring categories, unidimensionality, reliability and differential item function. Data from 624 persons with a diagnosis of painful diabetic polyneuropathy, small fibre neuropathy, and neuropathic pain associated with chronic low back pain, spinal cord injury, HIV-related pain, or chronic post-surgical pain was used for this analysis.

**Results:**

PD-Q demonstrated fit to the Rasch model after adjustments of scoring categories for four items, and omission of the time course and radiating questions. The resulting seven-item scale of pain qualities demonstrated good reliability with a person-separation index of 0.79. No scoring bias (differential item functioning) was found for this version.

**Conclusions:**

Rasch modelling suggests the seven pain-qualities items from PD-Q may be used as an outcome measure. Further research is required to confirm validity and responsiveness in a clinical setting.

**Electronic supplementary material:**

The online version of this article (doi:10.1186/s12883-017-0825-2) contains supplementary material, which is available to authorized users.

## Background

Pain is not only a multi-faceted sensory and emotional experience, but can present in different forms [1]. *Nociceptive* pain is considered to be the protective warning system to signal or avoid tissue damage [2], while *neuropathic* pain (NeP) represents a persistent pain resulting from damage to the nervous system [3]. PainDETECT (PD-Q) is a 9-item self-report screening questionnaire developed to detect NeP in conditions like chronic low back pain [4]. PD-Q measures 7 aspects of the quality of the pain experienced, the chronological pattern (*time course*), and whether or not the pain radiates. It is scored from 0 to 38, with total scores of less than 12 considered to represent nociceptive pain, 13–18 possible NeP, and >19 representing >90% likelihood of NeP (see Additional file 1: Supplementary Figure A for a sample of the form and associated scoring). The developers undertook classic psychometric testing in 392 persons with varied conditions including low back pain, post-herpetic neuralgia, painful polyneuropathy, osteoarthritis, visceral pain, and inflammatory arthropathies. They reported a sensitivity of 84% and specificity of 84% for NeP compared to a reference standard based on expert examination, and robust internal consistency amongst the 7 pain quality items. [4] The US English version of this tool has been employed to evaluate populations including osteoarthritis [5, 6], rheumatoid arthritis [7, 8], amyotrophic lateral sclerosis [9], neck and upper limb pain [10], and a cross-section of NeP conditions [11, 12].

### Rasch analysis

Rasch analysis is an approach aimed at understanding the measurement characteristics of an assessment. A key advantage of this type of analysis is if data produced by a measure like PD-Q fit the Rasch model, the ordinal scale measurements of individual test items (such as PD-Q’s *Never* to *Very Strongly* ratings) can be converted into interval-level scaling like 0 to 5 that can be credibly summed into total scores, with desirable measurement properties [13, 14]. Another key premise of Rasch modelling is invariance of the model across samples: meaning a Rasch-validated tool can be expected to measure the same way regardless of the population being studied [15, 16] because the assessment itself is validated, not the measurement characteristics for a specific population.

In contrast to traditional item response theory (IRT), Rasch analysis evaluates measurement characteristics using probability estimates, describing items as easy or difficult relative to the ability of the respondents [16–18]. For example, an item would be considered ‘easy’ if most respondents, even those with severe disease scored favorably on the item, and ‘difficult’ or ‘severe’ if only persons with mild disease scored favorably on the item. Persons and items are “*fit*” to this fixed model rather than developing a model around the data points [13]. The average difficulty/severity of the items is typically set to zero as a reference for this fitting process, and both item-level (difficulty or severity) and person-level estimates on the construct attempting to be measured (e.g., level of NeP) are standardized to *Z* scores [19]. The final key concept in Rasch theory is *unidimensionality;* that is, each scale or subscale represents a single characteristic or construct.

### Rasch analysis and painDETECT

The PD-Q utilizes a 0–5 adjectival scoring system for pain qualities instead of the dichotomous present/absent format often seen in screening tools. Since multi-level scoring is preferable for measuring health outcomes: [20] that is, longitudinally measuring change over time, it is possible PD-Q could serve this purpose [11]. If the current 0–5 scaling could be shown to demonstrate interval-level properties, or be transformed to provide interval-level measurement, it could support the use of the PD-Q as an outcome measure.

Moreton et al [5] conducted a Rasch analysis of the PD-Q on 135 subjects with osteoarthritis (OA) to consider its potential as an outcome measure and advocated omission of the *pain course* item for optimal model fit [5]. The remaining items (7 pain qualities plus *radiating*) demonstrated fit to the model, but the analysis of a single population with largely nociceptive pain suggested PD-Q may lack the precision to measure outcomes in persons with few or no features of NeP [5]. However, it is important to note participants were not physician-assessed to confirm the diagnosis of NeP, which was self-reported using PD-Q in 27% of the sample. Therefore, further Rasch analysis of PD-Q is warranted using a relatively large heterogeneous population with a range of physician-confirmed NeP diagnoses (including but not restricted to OA) in order to examine differential item function or (potential measurement bias) by diagnosis. The purpose of this study is to use Rasch analysis to assess whether painDETECT demonstrates measurement properties consistent with an outcome measure.

## Methods

### Participants

This study is a secondary data analysis of a previously published cross-sectional survey of the burden and costs for 624 patients with NeP [21, 22]. The NeP conditions examined in the study were painful diabetic peripheral neuropathy (pDPN), chronic lower back pain with NeP, spinal cord injury related NeP (SCI-NeP), small fiber neuropathy, human immunodeficiency virus related NeP, and post-trauma post-surgical pain.

### Variables of interest

Data from the NeP survey were compiled in Excel for demographic examination and imported to RUMM2030 version 5.1 (RUMM Laboratory Pty Ltd: Perth, Australia) for Rasch analysis. Demographic data included age, sex, and NeP diagnostic group (see Table 1); other person-level characteristics included in the analysis were summary scores from the physical and mental components scales (PCS and MCS) of the SF-12 [23] and pain severity and pain interference scores from the Brief Pain Inventory (BPI) [24, 25]. Variable selection originated from a rank-ordering exercise of six external experts in NeP from a network of clinicians and scientists working on the development of a **C**ore **O**utcome **M**easures for complex regional **PA**in syndrome **C**linical S**T**udies (COMPACT) [26]. Detailed and accessible description of the Rasch model and the application to scale analysis is published elsewhere for the interested reader [15–17, 27, 28].

**Table 1:** Demographics (including description and coding for person variables). N.B. Means presented are from raw scores, not categorized values. Key: NeP = neuropathic pain, SF = short form, PCS = Physical components summary, MCS = Mental components summary, BPI = Brief Pain Inventory

Person Variable	Coding	N** (%)
Age Sample mean age = 55.4 years Total N = 624	Under 20	1 (0.2)
	20–29 years	24 (3.8)
	30–39 years	43 (6.9)
	40–49 years	134 (21.5)
	50–59 years	199 (31.9)
	60–69 years	122 (19.6)
	70–79 years	73 (11.7)
	80 plus	28 (4.5)
**Sex**	Male	347 (55.6)
	Female	277 (44.4)
**NeP Diagnosis**	Chronic low back pain	103 (16.5)
	Post-Diabetic peripheral neuralgia	100 (16.0)
	HIV related pain	103 (16,5)
	Chronic post-surgical pain	106 (17.0)
	Small fiber neuropathy	110 (17.6)
	Spinal cord injury	97 (15.5)
**SF12 PCS** Mean PCS = 31 (100 = perfect health)	0–19	61 (10.0)
	20–39	446 (73.0)
	40–59	102 (16.7)
	60–79	2 (0.3)
	80–100	0
**SF12 MCS** Mean MCS = 42.5 (100 = perfect health)	0–19	22 (3.6)
	20–39	241 (39.4)
	40–59	288 (47.1)
	60–79	60 (9.8)
	80–100	0
**BPI pain severity** Mean =5.2	0–3 Mild	111 (18.1)
	4–6 Moderate	297 (48.4)
	7–10 Severe	206 (33.6)
**BPI pain interference** Mean = 5.6	0–3 Mild	163 (26.2)
	4–6 Moderate	244 (39.2)
	7–10 Severe	216 (34.7)

** some total results for individual tests may not add up to 624 if data from a particular scale were missing; percentages are reported for the total number of available data sets

Sample size for Rasch analysis can be calculated following Linacre’s rule-of-thumb formula of *n* = 20 x number of items or *n* = 250, whichever is larger [29]. Thus, for the 9 item PD-Q, a sample of 250 would be the minimum required to support the accuracy of estimates of item difficulty or severity [27, 29].

### Analysis plan

Statistical analyses mirrored those recommended by Tennant et al [19], incorporating the iterative step testing of Lundgren-Nilsson and Tennant [27], which included: 1) response distribution, 2) class interval distribution, 3) unidimensionality, 4) thresholds (including rescoring when required), 5) individual person fit, 6) local dependency, 7) differential item functioning (DIF), and 8) item fit to the scale. Unidimensionality is confirmed by a two-step process: 1) principal components factor analysis to identify the positive and negatively loading patterns of the items, and then 2) subsequent t-testing of the cluster of positively loading items against the negatively loading items. [19, 30] The theoretical basis of this strategy is if the scale is truly unidimensional, the groups will be have a positive *t*-test at *p* = 0.05 [19]. After these examinations, deletion of mis-fitting items was considered and the balance of statistical scale robustness and clinically important items were weighed [27]. Bonferroni corrections for multiple analyses were used when appropriate.

The person separation index (PSI) was calculated to estimate how many comparative groups could be determined within the sample, and whether the scale was sufficiently robust for group or individual comparisons [19, 31]. The PSI is also used as an indicator of measurement reliability. Finally, the person-item distribution was plotted to consider how well the persons in the sample match traits being measured by the scale, also known as the targeting of the scale to the sample. A more detailed summary of analyses and associated statistical tests can be found in Additional file 1: Supplemental Table A.

## Results

### Demographics

Six hundred and twenty-four data files from a published NeP burden of illness study were available for Rasch modelling [22]. Men accounted for 55.6% of the sample and mean age was 55.4 years (Table 1).

### Analysis of 9 item PD-Q

#### Distribution of responses

All levels of scoring were used for all items; a score of 0 on the tingling item was the least frequently endorsed, with only 28 of the 620 persons completing the item scoring themselves as *‘never’* for experiencing tingling or prickling sensations in the area of their pain. Furthermore, 400 persons (64%) described their pain as radiating (see Additional file 1: Supplementary Table B for the full listing of response distributions). Review of the class interval distribution using a 10-class interval structure recommended by RUMM2030 on the basis of sample size demonstrated high variability across the 10 class intervals. Alternate class interval structures were thus explored, and a 4 class interval structure was chosen, yielding more balanced groupings of 142–162 persons per item distributed across the class intervals.

#### Thresholds

**Fig. 1:**
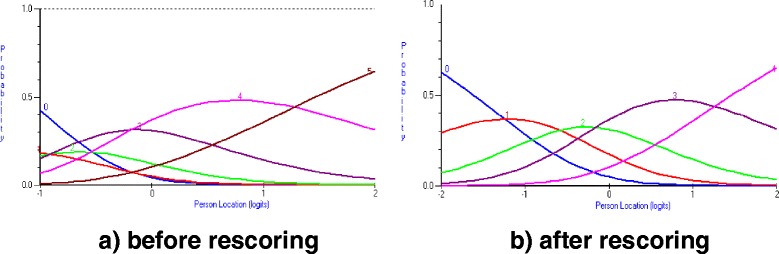
Category probability curves for the burning item. **a** before rescoring (**b**) after rescoring

A threshold indicates the point where there is a 50/50 probability of respondents choosing between any two adjacent score categories; thus the number of thresholds is always one less than the number of score categories. Initially, five items on the PD-Q (*burning, tingling, electric-shock, numbness* and *time course*) presented disordered thresholds by Rasch analysis. For example, responses to these questions on pain quality did not follow the same progression (from low scores to high scores) as the progression of severity of the person scoring (from low levels to high levels of NeP). This point is illustrated in Fig. 1a, where the probability of scoring a 0 out of 5 on the burning item overlapped the probability of scoring 1/5. Therefore, items were re-scored by collapsing response categories based on the category probability curves (see Fig. 1a & b for graphs representing *burning*) until the thresholds demonstrated sequential levels of severity. This resulted in a decrease in the number of response categories from 6 to 5 for the *burning, tingling*, *electric shock*, and *numbness* items. The *time course* item was rescored to combine pain attacks with and without pain between attacks. Refer to Table 2 for an illustration of how the scoring categories were collapsed for each disordered item.

**Table 2:** Rescoring Key

Item	Original Scoring 0 1 2 3 4 5
Burning	Re-scored 0 1 1 2 3 4
Tingling	Re-scored 0 1 1 2 3 4
Electric Shock	Re-scored 0 1 1 2 3 4
Temperature	Re-scored 0 1 1 2 3 4
Time course	**Original scoring 0 1 2 3**
	Re-scored 0 1 2 2

#### Fit to the rasch model

Once the thresholds were re-scored, initial fit analysis of PD-Q to the Rasch model revealed a significant chi-square value for item-trait interaction [χ^2^(27) = 84.3 *p* <0.000], suggesting when considered as a whole, the PD-Q did not fit the Rasch model. Therefore we proceeded to look at each aspect of fit to identify where the misfit was coming from, and if it could be addressed in the model.

#### Individual person fit

Relative to total PD-Q score, one case was designated as extreme (scoring far lower than would be expected), and was thus excluded from further analyses. Overall person fit statistics had a mean of *Z* = 0.06, suggesting the average scores were very close to what was expected with an acceptable SD = 0.83 (see Additional file 1: Supplementary Table A). Examination of the person-item threshold distributions for the total PD-Q showed statistically significant differences in NeP based on sex, age, PCS and MCS scores, BPI pain severity, and BPI pain interference (*p* <0.01 for all) but not NeP diagnosis (*p* = 0.09).

#### Response dependency

Analysis of PD-Q item residuals demonstrated high correlation between the *burning* and *tingling* items which may be the source of misfit to the Rasch model. Procedurally, it is recommended these items be treated as a single unit or testlet [32], during scoring calculations but not during administration of the assessment to the patient; [19] therefore *burning* and *tingling* were always considered together in all future analyses of overall fit.

#### Differential item function

**Fig. 2:**
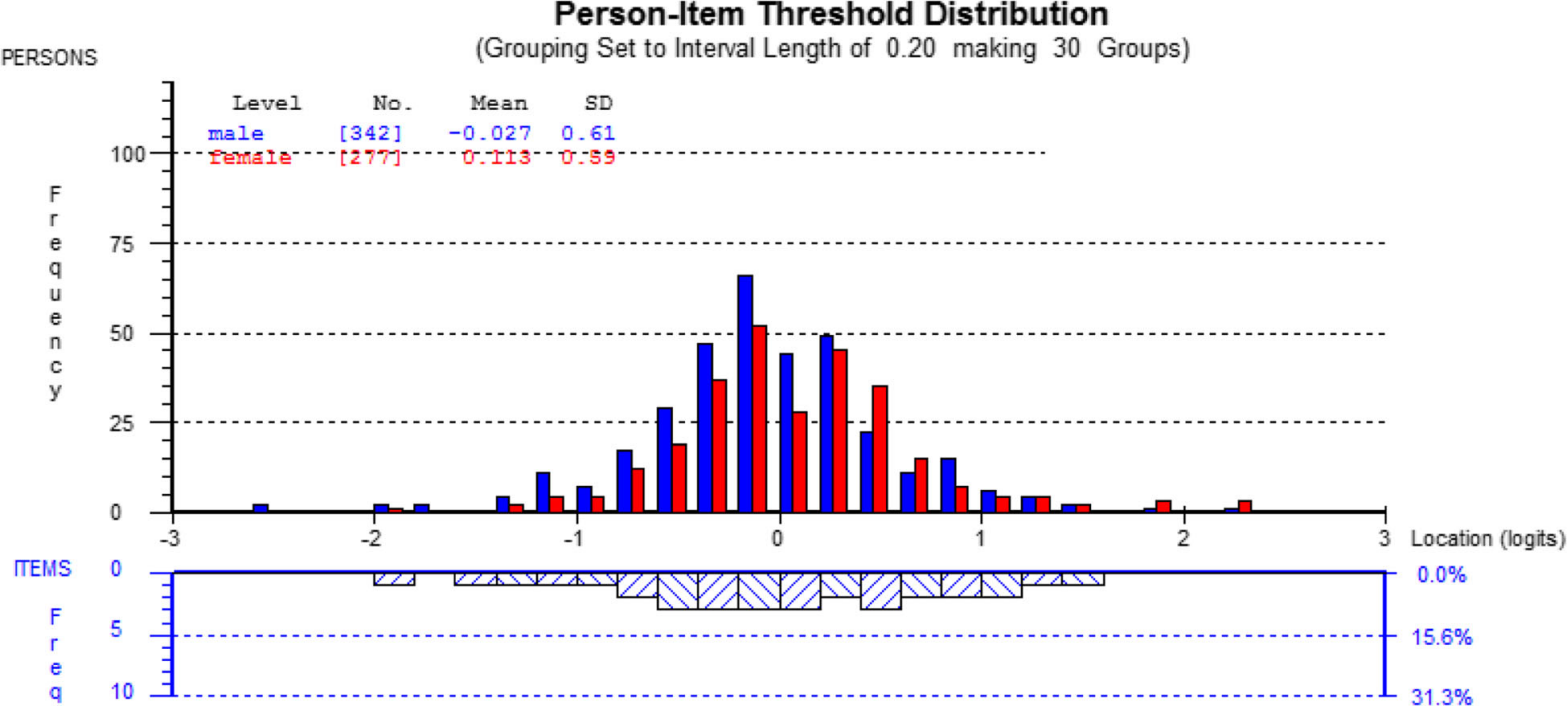
Person-Item Map grouped by sex. Key for Fig. 2: These dual histograms illustrated the relationship of the severity of NeP in the respondents (top) to the difficulty of the items (bottom). The logits scale on the x-axes of the graphs represents a standardized score where the mean severity or difficulty is set to 0, and one logit = one SD. The y-axis of the top histogram shows the probability of attaining the standardized score if you are a male vs. female; while the lower histogram shows the probability of endorsing a given score for a particular item

Differential item function describes the risk of systematic bias (uniform DIF) or random bias (non-uniform DIF). In this analysis, 4 PD-Q items showed uniform DIF. The *time course* was scored differently on the basis of age [*p* = 0.001] and BPI severity [*p* <0.001] and the *radiating*, *tingling* and *pressure* items were scored differently on the basis of NeP diagnosis [*p* <0.001 to *p* = 0.002]. For example, the DIF by NeP diagnosis in subjects with pDPN consistently scored lower than expected on the tingling item, while persons with SCI-NeP scored consistently higher than expected. It was interesting to note that despite no evidence of DIF on the basis of sex, the average PD-Q score was lower for men than for women when comparing the two groups (F_1,617_ = 8.32, *p* = 0.004): see Fig. 2 for a visual representation of the converted scores. In clinical terms, this suggests women in the sample truly had higher levels of pain than men, and did not just score themselves higher on the questionnaire.

#### Individual item fit

Individual items were reviewed for acceptable fit residuals of less than + 2.5 [19]. Of all items in the PD-Q, the *time course* item was flagged as not fitting with a fit residual value = +8.5, along with the *radiating* item with a fit residual of +3.2.

#### Scale and item re-appraisal

Because the *time course* and *radiating* items had unacceptably high fit residuals [19] which could influence other analyses (as seen in DIF), we excluded them from future iterations. This left the seven pain quality items as had been considered by other authors [4, 12]. After these revisions of the scale, it was necessary to recheck class interval distribution and unidimensionality indicators [13] for this 7-item outcome measure iteration.

### Analysis of an 7-item PD-Q outcome measure

With *burning* and *tingling* treated as a testlet, this 7 item PD-Q demonstrated fit to the Rasch model with item-trait interaction statistics of χ^2^ (12) = 20.4, *p* = 0.06 over three class intervals for ideal balance. This non-significant chi-square value indicates no difference, or unidimensionality. However, further exploration of the scale fit revealed DIF by NeP diagnosis for both the *burning/tingling* testlet and the *pressure* item. We therefore elected to combine the burning, tingling, and pressure items as a background ‘subtest’ to see if this DIF would cancel out across the scale as long as all 3 of these items were included on the scale [19]. This iteration produced item-trait interaction statistics of χ^2^ (10) = 16.9, *p* = 0.08 over 3 class intervals, again favoring unidimensionality. The PSI, an indicator of reliability, was 0.79, indicating sufficient reliability for group-level comparisons [31].

#### Person-item distribution

**Fig. 3:**
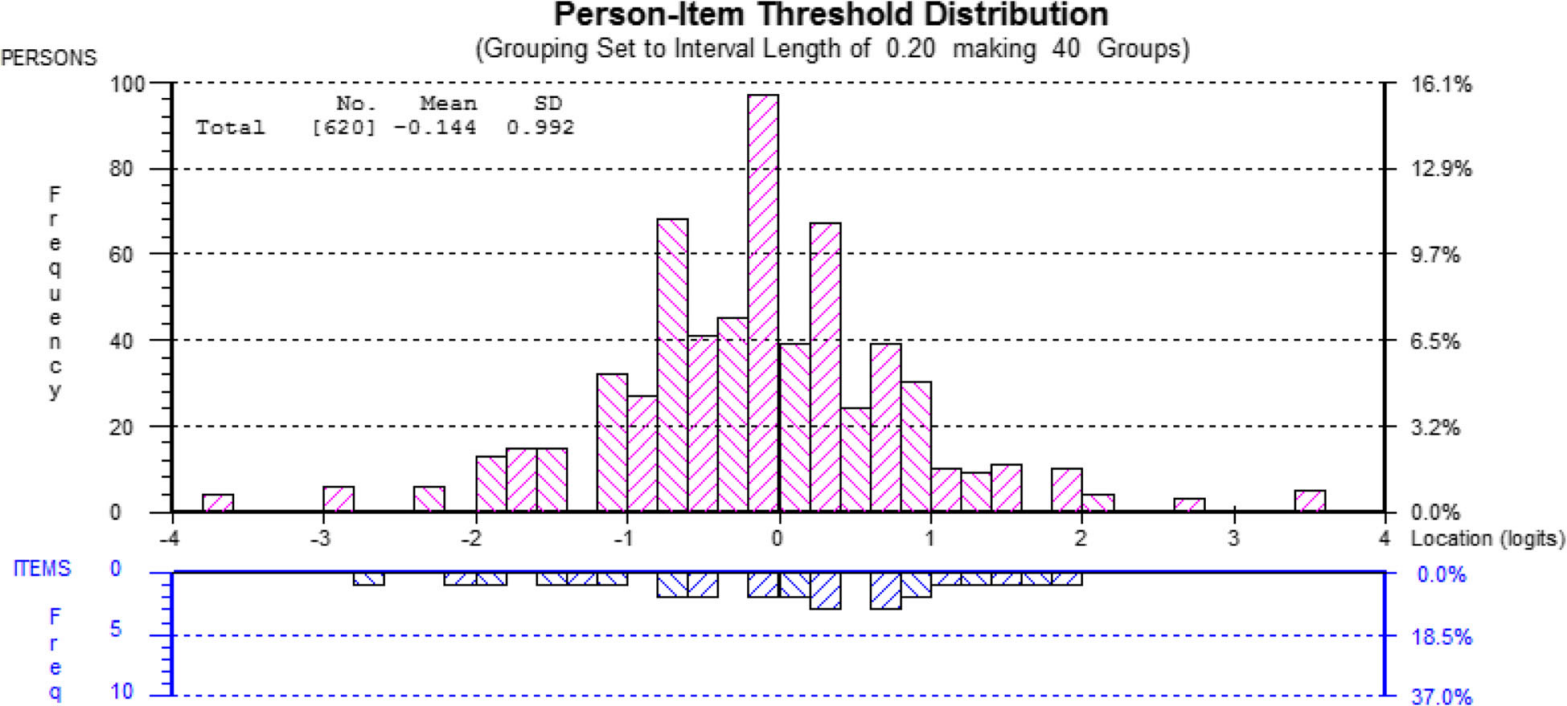
Person-Item threshold map. Key for Fig. 3: These dual histograms illustrated the relationship of the severity of NeP in the respondents (top) to the difficulty of the items (bottom). The logits scale on the x-axes of the graphs represents a standardized score where the mean severity or difficulty is set to 0, and one logit = one SD. The y-axis of the top histogram shows the distribution of standardized scores while the lower histogram shows the probability of endorsing a given score for a particular item

Person-item distribution is a graphical representation of how well the difficulty/severity of the items match the extent or level of the concept of interest (e.g. NeP) in the subjects. Figure 3 illustrates that few persons (represented by bars in the top histogram) fell outside the range of severity measured by the PD-Q items (represented by the bars in the bottom histogram). Analysis of variance on the standardized (Z) scores by NeP diagnosis (F_(5)_ = 2.02, *p* = 0.07) indicated no difference in mean PD-Q scores between diagnostic groups.

#### Unidimensionality

Analysis of the 7 item PD-Q met the unidimensionality criterion, as indicated by the proportion of tests finding a difference found between split halves of the scale; this was less than 5% (*p* = 0.028) [30]. This procedure partitions the scale items using factor analysis into positively and negatively loading items, and then t-tests these 2 item groups for each person to ensure they are not different, supporting the hypothesis all items are measuring a single trait [32]. See Table 3 for a summary of all fit criteria for both the original 9 item and proposed 7item outcome measure version of PD-Q. See also Additional file 1: Supplementary Figure A for the item map of the final 7-item version.

**Table 3:** Summary statistics for all analyses

	Unidimensionality	Item location	Person location	Item fit residual	Person fit residual	Item Trait Interaction	PSI
9 item PD-Q (original)	*p* = 0.065 95%CI 0.048–0.082	Z = 0.00 SD = 0.37	Z = 0.14 SD = 0.69	Z = 0.77 SD = 3.65**	Z = -0.28 SD = 1.26**	*χ* ^2^ (27) = 104.8 *p* <0.000**	0.77
7 item PD-Q	*p* = 0.028 95%CI 0.011–0.045	Z = 0.00 SD = 0.34	Z = -0.11 SD = 0.99	Z = 0.36 SD = 3.39**	Z = -0.39 SD = 1.20**	*χ*2 (10) = 16.78, *p* = 0.079	0.79

** denotes results which demonstrate misfit

## Discussion

### Summary of findings

We conducted a Rasch analysis of PD-Q to evaluate its measurement properties as an outcome measure for possible use research and clinical practice.

#### Results in light of the existing literature

A Rasch analysis of this tool was completed previously in a more homogeneous population: [5] the results of our study, based on physician-confirmed diagnosis of one of 6 NeP conditions, further demonstrate support for use of PD-Q as an outcome measure.

Despite the dataset having more men than women (ratio 1.25:1), no DIF was found based on sex. However, women achieved higher standardized scores than men on average, (Fig. 2), suggesting they had higher levels of NeP, which aligns with known population trends in chronic pain [33, 34].

We were able to demonstrate a 7-item version of the PD-Q has the potential for use as an outcome measure for NeP qualities, as it was able to fit the Rasch model by representing a single construct (is unidimensional); thus also supporting the Rasch assumption of invariance, or universal measurement properties across populations. Additionally, invariance is further supported by the lack of differences (*p* = 0.07) seen among NeP diagnoses in person-item thresholds. Further, it reflects factor analysis presented by both the original developers of the tool which showed all 7 pain qualities loading on a single factor [4] and previous analysis of this data set supporting good internal consistency of a 7-item PD-Q [12].

In the Rasch paradigm, reliability is represented by the person-separation index. Our value of 0.79 fell into the ‘Good’ range [19]. In contrast, Moreton et al [5] reported better discriminative function as demonstrated by a PSI value of 0.83 [31] for their 8-item iteration of PD-Q in a potentially underpowered sample.

While our study replicated the findings of Moreton et al [5] in excluding the *time course* item and identification of local dependency in the *burning* and *tingling* items, there is an important difference. Their study examined PD-Q in a population of persons with knee OA, reporting 27% of the group (total *n* = 192) as having likely NeP; scores ranged from 8 to 18 with an average score of 13 (out of a maximum score of 38). However, the identification of NeP was based entirely on self-report measures, and was not confirmed by physician diagnosis. In contrast, our population of 624 persons all had physician confirmed diagnoses of NeP with an average PD-Q score of 20.4 (range 1–37). Accordingly, Moreton et al [5] reported PD-Q was not ideally targeted to their sample of persons with largely nociceptive pain resulting from OA, while our results, based on a larger sample and broader range of scores, demonstrated excellent targeting of the PD-Q items across the range of NeP severity represented in our population (see Fig. 3).

### Fit challenges and implications for practice

Because PD-Q was developed as a NeP screening tool and not an outcome measure, we anticipated a lack of fit to the Rasch model in its current form. In fitting the data to the Rasch model, the ordering of categories on certain items did not reflect corresponding increases in the amount NeP qualities the items were intended to measure: thus it was necessary to collapse the scoring categories on five of the original nine items. This rescoring procedure corrected all disordered pain quality items, suggesting respondents had trouble distinguishing between *‘hardly noticed’* and *‘slightly’.* In other words, the probability that persons with similar amounts of NeP would choose one description or score over another, was not predictable.

Overall, our results suggest a 7-item PD-Q may be appropriate for comparison of outcomes across populations, with rescoring of the *burning, tingling, electric shock* and *numbness* items. Scoring of the Rasch-endorsed format would potentially alter the current total score of 38 for 9 items to 31 for 7 items. This allows representation of the ordinal responses of the PD-Q pain quality items as a linear scale [35].

#### Implications for clinical practice

Rasch analysis suggested exclusion of the pictorial *time course* and dichotomous *radiating* item from summed scores. This in no way suggests altering the current 9 item form for its validated use as a screening tool for NeP, yet suggests they may not be as important when tracking outcomes of the PD-Q (i.e., NeP characteristics over time). Further research should seek to replicate these findings and consider opportunities to develop a Rasch scoring conversion table for easy transformation of clinically derived ordinal PD-Q scores from the original questionnaire to interval level scores to support accurate longitudinal monitoring to inform clinical care and research.

### Implications for research

#### Study limitations

It is not known if there is selection bias inherent in the initial data collection for the NeP survey that would influence PD-Q scores. A potential bias arises from the restrictions of the RUMM2030 software, which allows only 7 person-level factor to be used to describe and categorize the participants. The decision of which factors to include was made by the research team, and may have restricted the opportunity to discover DIF for PD-Q items related to other important population characteristics. However, our decisions were informed by a consensus exercise developed from a core measurement set for clinical trials in other pain conditions [26].

While the sample size of 624 NeP subjects was sufficient for analysis, there is a risk this larger sample is powered to find very small differences, making the target of a non-significant chi-square value for item-trait interaction increasingly stringent.

## Conclusion

The Rasch model supported the acceptance of a shorter 7-item pain quality outcomes measure, excluding the *time course* and *radiating* items from the original measure. This outcome measure conversion of PD-Q may prove useful for clinicians who wish to accomplish the dual goal of screening for NeP at baseline, and tracking changes in pain qualities over time; the dual purpose could also reduce the overall burden of measurement in clinical trials. Future research is warranted to confirm the validity and responsiveness of this Rasch-informed 7-item outcome measure in a clinical setting.

## Additional file

Additional file 1:
**Table SA.** Analysis plan summary and Rasch definitions. **Table SB.** Response distribution. **Figure SA.** Item map. (DOCX 30 kb)

## Abbreviations

BPI: Brief pain inventory
DIF: Differential item function
MCS: Mental components summary
NeP: Neuropathic pain
OA: Osteoarthritis
PCS: Physical components summary
pDPN: Painful diabetic peripheral neuropathy
PD-Q: PainDETECT questionnaire
PSI: Person separation index
SCI-NeP: Spinal cord injury related NeP
SF: Short form
