# Supplementary material for: Measurement properties of painDETECT: Rasch analysis of responses from community-dwelling adults with neuropathic pain
Source: BMC Neurol. 2017 Mar 4;17:48. doi: 10.1186/s12883-017-0825-2 (PMC5336691; doi:10.1186/s12883-017-0825-2)

Supplementary Figure A: (see attached pdf for sample copy of the PD-Q questionnaire)

Supplementary Table A: Analysis plan summary and Rasch definitions

| **SCALE TRAIT** | **METHOD OF ANALYSIS** | **HYPOTHESIS/**  **EVALUATIVE CRITERIA** |
| --- | --- | --- |
| **Partial credit**  **(PC) vs. rating**  **scale (RS) parameters** | Log-likelihood ratio | If RS parameters are satisfied (ratio test is non-significant), use RS format; if not, choose PC version |
| **Response distribution** | Frequency plot of actual responses across all items and response options | Check that each possible category of item/response is endorsed; ideal to have at least 5 cases in each |
| **Thresholds** | Graphic representation and plots of probability | Ordered progression of thresholds from less to more of the trait |
| **Person fit** | Chi-square, fit residual transformed to a standardized (Z) score | Mean 0, SD up to 1 if data fit the Rasch model; reflects the divergence between expected and actual values looking across all items scored by an individual person |
| **Item fit** | Chi-square, fit residual transformed to a  Z-score | Mean 0, SD up to 1 if data fit the Rasch model; reflects the divergence between expected and actual values looking across all persons for a given item |
| **Item trait interaction** | Chi-square probability | p>0.05 tests whether the items are working as expected across the class intervals for the trait |
| **Individual person fit** | Chi-square analysis | Values should fall within ±2.5 if the data fit the Rasch model |
| **PSI** | Cronbach’s alpha | 0-1; values over 0.70 allow for group comparison, over 0.85 for individual comparisons of summed scores |
| **Local dependency** | Correlation analysis of item residuals | Look for correlations of over 0.2 to 0.3 |
| **DIF** | Item characteristic curves (IC curves); ANOVA of item residuals | IC curves plotted by person characteristics (such as age, sex, NeP diagnosis,) and p>0.05 for between groups ANOVA reveal DIF |
| **Unidimensionality** | Each subscale is partitioned using principal component factor analysis and subsequently t-tested | There will be no significant difference between the two partitioned pieces of the subscale |

NeP=neuropathic pain; ANOVA= analysis of variance; DIF=differential item functioning; PSI= person separation index

**Supplementary Table B. Response distribution**

| PDQ item | Number of endorsements | | | | | |
| --- | --- | --- | --- | --- | --- | --- |
|  | 0 | 1 | 2 | 3 | 4 | 5 |
| Radiating | 224 | 400 | n/a | n/a | n/a | n/a |
| Time course | 132 | 246 | 72 | 162 | n/a | n/a |
| Burning | 45 | 33 | 62 | 161 | 213 | 100 |
| Tingling | 28 | 37 | 74 | 182 | 208 | 91 |
| Light touch | 123 | 116 | 142 | 129 | 72 | 40 |
| Electric shock | 67 | 44 | 89 | 153 | 171 | 98 |
| Temperature | 151 | 146 | 107 | 120 | 67 | 32 |
| Numbness | 47 | 29 | 89 | 122 | 181 | 123 |
| Pressure | 78 | 88 | 108 | 153 | 128 | 60 |

N.B. The total number of endorsements for any item may vary slightly based on missed questions.

**Supplementary Figure A. Item map**

This illustrates the difficulty of each response option for every item mapped against the ability (or amount of the construct of interest) of the persons in the sample. Person abilities are represented in the histogram on the left side; item difficulty are coded as I0003.1 for response option one on item 3 (*burning*=0), while I0003.2 for response option 2 on item 3 (*burning*= 1 or 2) [remember: this graphic represents the rescored items retained in the final model, and does not include the deleted radiating and time course items]. Both the difficulty and person abilities have been calculated relative to the standardized scores of logits represented by the central column of numbers from -4.00 to 4.00.

An interpretation of this graphic is, for example, persons with the lowest levels of NeP are most likely to score zero (response option 1) for items 3, 4, and 8 on PDQ (the *burning, tingling* and *numbness* items) [seen in the bottom row of items].


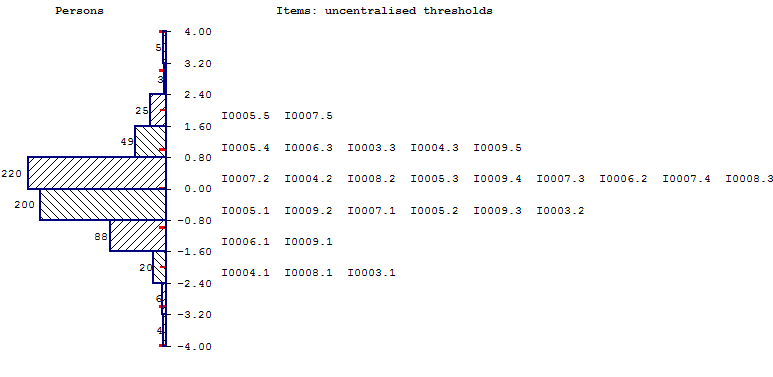

Supplement: Additional file 1: — Table SA. Analysis plan summary and Rasch definitions. Table SB. Response distribution. Figure SA. Item map. (DOCX 30 kb) [file 12883_2017_825_MOESM1_ESM.docx]
